# *Klebsiella pneumoniae* vaccines: Evolving the blueprint from traditional platforms to mucosal and nanoscale delivery

**DOI:** 10.1016/j.mtbio.2026.102919

**Published:** 2026-02-13

**Authors:** Jinping Hu, Yiyi Xie, Weiqi Guan, Linlin Huang, Xin Li

**Affiliations:** a Shandong Province University Clinical Immunology Translational Medicine Laboratory, The First Affiliated Hospital of Shandong First Medical University & Shandong Provincial Qianfoshan Hospital, Jinan, 250014, PR China; b School of Clinical and Basic Medical Sciences, Shandong First Medical University & Shandong Academy of Medical Sciences, Jinan, 250117, PR China

**Keywords:** *Klebsiella pneumoniae*, Carbapenem resistance, Nanovaccine, Vaccine development, Clinical translation

## Abstract

*Klebsiella pneumoniae* (*KP*) is a critical global health threat due to carbapenem-resistant and hypervirulent strains, necessitating effective vaccines amid declining antibiotic efficacy. This review provides a timely and systematic analysis of the current *KP* vaccine landscape. We first outline the epidemiology of classical and hypervirulent *KP* (*hvKP*) and the corresponding protective host immunity, establishing a foundation for vaccination. A detailed survey of key antigenic targets, including polysaccharides and proteins, is presented. The core of this review critically assesses the spectrum of vaccine platforms, from traditional whole-cell and subunit vaccines to nanovaccine platforms. Distinctively, we place a dedicated focus on two transformative frontiers: nanovaccines—such as outer membrane vesicles (OMVs) and biosynthetic glycoconjugate nanoparticles—and mucosal vaccination strategies, emphasizing their unparalleled potential to block infection at the primary site of colonization. By synthesizing cutting-edge preclinical advances and addressing persistent translational challenges, this review serves as a timely and comprehensive resource, offering valuable insights to guide the future development of effective immunization strategies against this formidable pathogen.

## Graphical abstract

**Figure:**
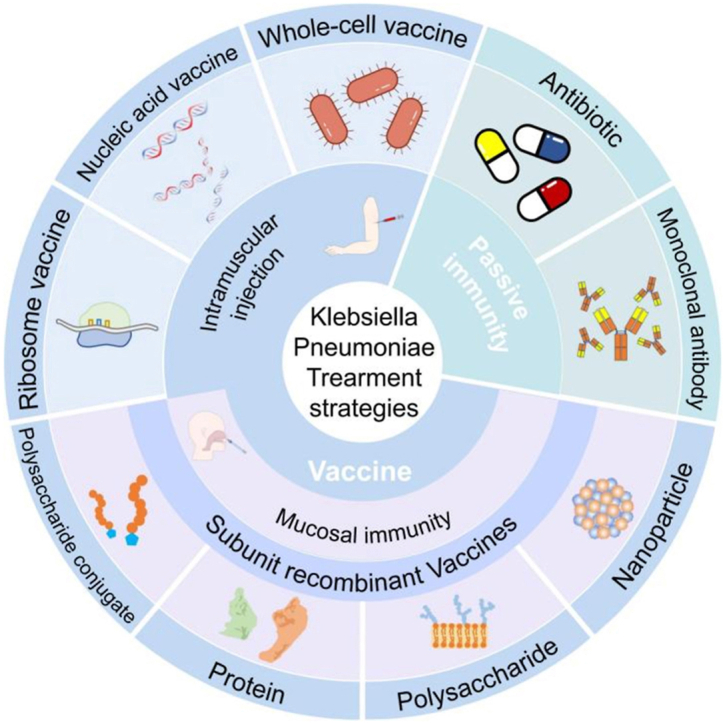

## 1 Introduction

*Klebsiella pneumoniae* (*KP*) is a Gram-negative opportunistic pathogen. It represents a leading cause of nosocomial infections, owing to its environmental adaptability and propensity to colonize human mucosal surfaces [1]. The clinical impact of *KP* is further intensified by the global spread of carbapenem-resistant *KP* (CR-*KP*) and hypervirulent *KP* (*hvKP*) [2]. CR-*KP* rapidly propagate resistance genes through mobile genetic elements, giving rise to multidrug-resistant (MDR) clones [3]. Meanwhile, *hvKP* strains often harbor virulence plasmids that enable invasive infections even in immunocompetent hosts [4]. In response to this urgent public health threat, the World Health Organization (WHO) has classified CR-*KP* as a priority "critical" pathogen [5].

Current therapeutic options are increasingly limited. Traditional β-lactam and carbapenem antibiotics are failing due to advanced resistance mechanisms, while passive immunization approaches, such as monoclonal antibodies (mAbs), face challenges related to antigenic diversity and rapid bacterial evolution [6]. These constraints underscore the need for effective vaccines as strategic tools to combat antimicrobial resistance (AMR). Vaccines can elicit active immunity against conserved antigens—such as OmpK36 and MrkA—or broad-spectrum epitopes, engaging both humoral and cellular immune mechanisms to confer durable protection, even against drug-resistant strains [7]. Although no licensed *KP* vaccine is yet available, the WHO has emphasized its development as a critical priority. The integration of genomics, immunomics, and synthetic biology holds promise for accelerating the translation of promising vaccine candidates from preclinical research to clinical application [8].

The rational design of a broadly effective *KP* vaccine faces two core challenges. First, the pathogen exhibits significant diversity in its surface antigens, particularly the capsular polysaccharide (CPS, also known as K-antigen) and the lipopolysaccharide (LPS) O-antigen [9]. Second, *KP* shows a strong propensity to initiate infection at mucosal sites [10]. To address these challenges, this review focuses on two complementary innovative strategies: nanovaccine platforms and mucosal vaccination. Nanovaccines utilize engineered carriers that enable multivalent antigen presentation, enhance antigen stability, and facilitate efficient delivery to immune cells [[11], [12], [13], [14]]. Mucosal vaccination, especially via the respiratory route, aims to establish a first line of defense. It works by inducing local secretory immunoglobulin A (SIgA) and tissue-resident memory (TRM) cells to block bacterial colonization before systemic invasion occurs [14,15].

**Fig. 1:**
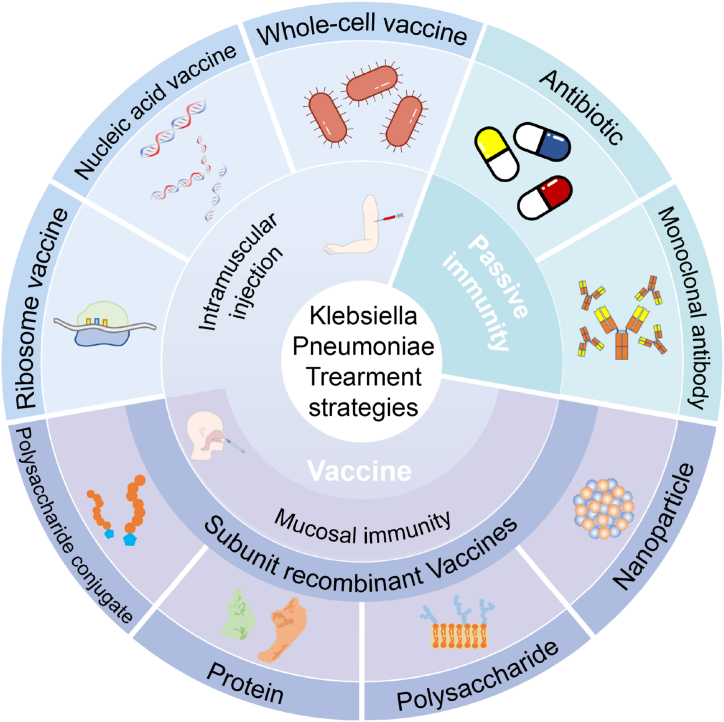
Landscape of current vaccine and immunotherapeutic strategies against *Klebsiella pneumoniae*. Created with Biorender.com.

This review systematically examines the epidemiological characteristics and major target antigens of *KP*, and highlights the latest advancements in prevention and treatment strategies (Fig. 1). We discuss the mechanisms and protective efficacy of various *KP* vaccine platforms, highlighting the potential of mucosal delivery systems for preventing *KP* colonization and infection. Furthermore, we synthesize lessons from recent clinical trials, analyze persistent translational challenges, and outline future research directions aimed at eliciting cross-protective immunity. Ultimately, this review aims to provide a comprehensive scientific foundation to guide the development of effective immunization strategies against this formidable pathogen.

## 2 Epidemiology of *Klebsiella pneumoniae*

*KP* poses a dual threat to global health through two primary pathotypes: classical *KP* (*cKP*) and *hvKP*. The emergence of hybrid strains that combine MDR and hypervirulence exacerbates this challenge [16,17].

*cKP* is a leading cause of nosocomial infections, particularly in immunocompromised individuals. These infections commonly manifest as hospital-acquired pneumonia, urinary tract infections, and bacteremia [18]. The clinical significance of *cKP* stems from its strong propensity to acquire resistance genes. This process often culminates in MDR, extensively drug-resistant (XDR), and CR-*KP* clones [19]. Predominant lineages, such as sequence type 258 (ST258) and 512 (ST512) in Europe/Americas and ST11 in Asia, drive the global spread of CR-*KP* [20,21]. Mortality from CR-*KP* is significantly influenced by host baseline characteristics. Therefore, patient risk profiles—rather than carbapenem resistance alone—may drive outcomes, highlighting the limitations of antibiotic-focused strategies [22].

In contrast, *hvKP* causes severe invasive infections in healthy individuals [23]. Examples include liver abscesses, necrotizing fasciitis, endophthalmitis, and metastatic complications [4]. Genetically, *hvKP* is distinguished from *cKP* by the presence of specific virulence plasmids, such as pK2044 and pLVPK, which carry key virulence genes including *iuc* (involved in aerobactin siderophore biosynthesis), *peg-344* (a metabolic transporter of unknown function), and *rmpA/rmpA2* (regulators of capsule hyperproduction) [24]. Although *hvKP* is traditionally antibiotic-susceptible, researchers now observe an alarming convergence of resistance and virulence. Through plasmid exchange, *hvKP* can acquire resistance plasmids from *cKP*, giving rise to hybrid strains [25]. Current evidence suggests that such hybrids may not retain full hypervirulence [26], yet ongoing genetic exchange between strains warrants close monitoring [27].

## 3 Surface antigen components of *Klebsiella pneumoniae*

### 3.1 Surface polysaccharides: dominant protective antigens

#### 3.1.1 Capsular polysaccharide (CPS)

CPS is a negatively charged polymer composed of repeating oligosaccharide units that exhibits considerable structural diversity. As the primary virulence factor of *KP*, CPS forms a protective barrier that suppresses phagocytosis, enhances tissue adhesion, and promotes biofilm formation. These functions collectively heighten resistance to antibiotics and host immunity, a process essential for bacterial immune evasion and colonization [28,29]. The *hvKP* capsules consist of strain-specific CPS, classified into K antigens (e.g., K1, K2, up to K78) [1]. Virulence varies substantially among serotypes, with K1, K2, K16, and K20 strains being particularly virulent [30]. To date, over 100 distinct capsular serotypes have been identified, with K1 and K2 being the most prevalent [31].

CPS biosynthesis is directed by a highly conserved gene cluster—including *wzi*, *wza*, *wzb*, *wzc*, *gnd*, *wca*, *cpsB*, *cpsG*, and *galF*—which coordinately regulates its assembly, export, and surface attachment [32,33]. The virulence of *KP* is strongly correlated with capsular thickness and mucoid phenotype. Notably, *hvKP* produces thicker capsules than *cKP*—a trait regulated by RmpA/RmpC proteins [34,35]—and its hypermucoviscosity phenotype, especially prominent in K1 and K2 serotypes, is genetically linked to the *rmpA* gene [36]. Collectively, these attributes underscore the critical role of CPS in immune evasion and pathogenicity of *KP*.

#### 3.1.2 Lipopolysaccharide (LPS)

LPS, also referred to as endotoxin, is composed of three structural domains: O-antigen, core oligosaccharide, and lipid A. These components are governed by the *wb*, *waa*, and *lpx* gene clusters, respectively [[37], [38], [39]]. In contrast to the diversity of K-antigens, O-antigen serotypes are fewer, comprising nine major types and over five subtypes [40]. Clinically, serogroup O1 predominates among *KP* isolates; together, O1, O2, O3, and O5 account for approximately 75-100% of cases [38], with most antibiotic-resistant and hypervirulent strains expressing O1 or O2 antigens [41]. The O-antigen plays a critical role in virulence: its full-length form confers resistance to complement-mediated killing, while truncation or loss increases susceptibility—even in encapsulated strains [42]. Mechanistically, the O-antigen prevents insertion and polymerization of the membrane attack complex (MAC) component C9 into the bacterial membrane [43].

### 3.2 Protein virulence factors: conserved candidate vaccine antigens

**Fig. 2:**
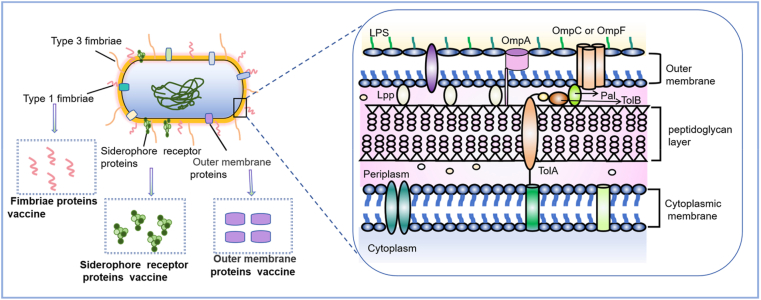
Schematic representation of key protein virulence factors on the surface of *KP*. Created with Biorender.com.

Protein-based virulence factors represent a strategic avenue for developing serotype-independent vaccines against *KP*. These highly conserved targets are primarily fimbriae, siderophore receptor proteins (SRPs), and outer membrane proteins (OMPs) (Fig. 2).

Fimbriae mediate critical early steps in *KP* pathogenesis, primarily facilitating host cell adhesion and biofilm formation [[44], [45], [46]]. Among them, type 1 and type 3 fimbriae have been extensively studied. Their major structural subunits are encoded by conserved gene clusters: type 1 fimbriae are encoded by the *fim* gene cluster (including *fimA*, *fimH*, *fimF*, and *fimG*) [47,48], while type 3 fimbriae are regulated by the *mrkABCD* operon [1]. Notably, due to its exceptional sequence conservation, the major subunit of type 3 fimbriae, MrkA, has become a leading antigenic target in current fimbria-based universal vaccine development [49,50].

SRPs are outer membrane transporters that recognize and internalize iron-bound siderophores under iron-restricted conditions [51,52]. *KP* strains commonly secrete one or more siderophores, notably enterobactin, salmochelin, yersiniabactin, and aerobactin. Accordingly, dedicated SRPs—FepA, IroN, FyuA, and IutA—serve as the cognate transporters for each corresponding siderophore [53].

OMPs are pivotal determinants of *KP* pathogenesis, critically influencing host immune modulation and AMR. Functionally, OMPs can be categorized into several groups, notably porins (e.g., OmpK35, OmpK36, OmpK37, OmpK38, PhoE, OmpK26), structural proteins (e.g., OmpA), and outer membrane lipoproteins (e.g., peptidoglycan-associated lipoprotein Pal, and the mucopeptide lipoprotein LppA) (Fig. 2) [54]. The dysregulation of OMPs, especially porins, is a central mechanism driving the pathogenicity of MDR strains [55,56]. For instance, amino acid insertions in the third extracellular loop (L3) of OmpK36 can occur. These insertions constrict the pore channel, reduce antibiotic permeability, and consequently enhance resistance to carbapenems [57].

Collectively, these surface antigens form the cornerstone of *KP* vaccinology. CPS and LPS O-antigen are the targets for serotype-specific and broad-spectrum polysaccharide-based vaccines, respectively [58]. Conversely, conserved protein antigens like fimbrial subunits and OMPs are pursued to develop universal vaccines [59]. The subsequent sections evaluate how different vaccine platforms leverage these targets to induce protective immunity.

## 4 Treatment strategies

Current therapeutic approaches for *KP* infections encompass pharmacotherapy (antibiotics), passive immunotherapy (mAbs), and active immunization (vaccines). While antibiotics remain the first-line clinical intervention, advances in biotechnology have positioned active immunization—particularly inhalable vaccines designed to elicit mucosal immunity—as a highly promising strategy with considerable therapeutic potential.

### 4.1 Antibiotic treatment

Antibiotic regimens for *KP* infections primarily rely on rapid bactericidal agents such as carbapenems, other β-lactams (e.g., cephalosporins), polymyxins, tetracyclines, fosfomycin, and cefiderocol [60]. Among these, carbapenems (e.g., imipenem and meropenem) represent the most potent and broad-spectrum β-lactam antibiotics, playing a critical role in *KP* management. They exert their effects by inhibiting penicillin-binding proteins (PBPs), thereby disrupting cell wall synthesis and leading to bacterial lysis [61].

A major contributor to *KP* antibiotic resistance is biofilm formation—structured microbial communities embedded in extracellular polymeric substances (EPS) [62]. Biofilms protect bacteria from host immune responses and antimicrobial penetration [58]. Strong biofilm-producing *KP* strains exhibit significantly elevated levels of extracellular DNA (eDNA), proteins, and EPS components compared to weak producers [59]. Interestingly, levofloxacin treatment has been shown to stimulate EPS production (exoproteins and exopolysaccharides), forming diffusion barriers that reduce antibiotic penetration and amplify resistance [63].

Despite the absence of antibiotics that specifically target biofilms, combination therapy has emerged as an effective strategy to mitigate this challenge. Studies demonstrate that drug combinations can synergistically enhance bactericidal activity and suppress biofilm development, thereby countering both resistance and heteroresistance—a state in which susceptible subpopulations exhibit transient antibiotic tolerance [58,60]. For instance, the polymyxin-meropenem combination not only exhibits potent bactericidal effects but also suppresses the emergence of polymyxin-resistant subpopulations [64].

In summary, combining conventional antimicrobials with agents that employ distinct mechanisms of action—including alternative anti-biofilm compounds—may yield synergistic effects against *KP* biofilms. Tailored antibiotic combinations represent a promising avenue to overcome biofilm-mediated resistance, enhance treatment efficacy, and combat MDR *KP* infections.

### 4.2 Passive immunization

Passive immunization confers immediate immune protection through the transfer of pre-formed pathogen-specific antibodies or immune cells, independent of the host's own immune response [65]. The increasing prevalence of MDR strains, particularly CR-*KP*, has spurred the development of pathogen-specific mAbs as a promising therapeutic alternative.

Antibody-based therapies offer several advantages over conventional antibiotics, including: (1) longer half-life; (2) highly specific pathogen targeting that minimizes disruption to commensal microbiota; and (3) multifunctional capabilities such as toxin neutralization, opsonophagocytic killing (OPK), and enhanced clearance by immune effector cells [66]. Current mAb development is focused on key antigenic targets including LPS, CPS, and non-polysaccharide epitopes, with the goal of optimizing therapeutic strategies against drug-resistant *KP* infections.

#### 4.2.1 Monoclonal antibodies targeting lipopolysaccharide O-antigen

A series of affinity-matured human mAbs derived from peripheral IgM^+^ and IgA^+^ memory B cells and intestinal plasmablasts have been developed to target the LPS O-antigen of *KP*. These mAbs demonstrated broad *in vivo* cross-reactivity and conferred protection against multiple clinically relevant *KP* serotypes [67]. For instance, the humanized mAb A1102, derived from a murine parent antibody recognizing a conserved O-antigen epitope, provided significant protection in galactosamine-sensitized mice challenged with live bacteria. Moreover, even at low-dose, A1102 protected endotoxin-sensitive rabbits against lethal ST258 infection [68].

Pennini et al. identified a human anti-O1/O2 IgG1 mAb that, when co-administered with antibiotics, significantly improved survival in mice infected with MDR-*KP* [69]. Cross-reactivity has also been observed between *KP* O3 and O5 serotypes, which produce mannose-rich O-antigens distinguished by terminal methyl (O3) or methyl phosphate (O5) modifications [70]. Further supporting this potential, two mAbs (O3a and O3b) generated against O3a: K11 strain LPS showed broad specificity against O3 subtypes and strong binding to the immunizing strain, together highlighting the feasibility of broad-spectrum immunotherapies even against highly resistant *KP* [71].

#### 4.2.2 Monoclonal antibodies targeting capsular polysaccharide K-antigen

The CPS of *KP* serves as a key virulence factor and a source of antigenic diversity. mAbs targeting conserved capsular epitopes represent a promising therapeutic strategy. For example, most *hvKP* strains express K1-CPS, against which mAbs 4C5 (IgG1) and 19A10 (IgG3) demonstrated protective efficacy in murine sepsis and pneumonia models [72].

CR-*KP* of ST258 represents a serious public health threat. mAbs targeting CPS have emerged as valuable therapeutic options for such infections [73]. A murine IgG3 mAb targeting *wzi1*54 CR-*KP* isolates showed superior functional efficacy over an IgG1 variant, including enhanced antigen-binding affinity, complement-dependent bactericidal activity, and neutrophil-mediated killing at lower concentrations [74]. Similarly, two IgG mAbs—17H12 and 8F12—isolated from mice immunized with CR-*KP*, mediated bacterial killing through biofilm disruption, complement activation, neutrophil extracellular trap (NET) formation, and enhanced opsonophagocytosis by neutrophils and macrophages [73]. These findings offer new avenues for clinical intervention against *KP*.

#### 4.2.3 Monoclonal antibodies targeting non-polysaccharide antigens

MrkA, the major structural subunit of type III fimbriae, has been explored as a target for serotype-independent mAbs. *In vitro*, anti-MrkA mAbs enhanced OPK while reducing bacterial loads in a murine pneumonia model [49]. These mAbs exhibited broad cross-reactivity across diverse *KP* clinical isolates of various O-serotypes, supporting MrkA as a target for antibody-mediated strategies against infection and biofilm formation [50]. Likewise, murine mAbs targeting the MrkD adhesin demonstrated protective efficacy in animal models [75].

The principal advantage of passive immunization lies in delivering immediate immunity irrespective of host immune status—thus rendering it particularly suitable for immunocompromised patients susceptible to opportunistic *KP* infections. Although protection is transient, passive immunization is gaining traction in *KP* management. Future efforts should focus on: (1) optimizing mAbs through advanced antigen screening and epitope refinement, and (2) exploring synergistic mAb combinations to improve therapeutic outcomes.

### 4.3 Active immunization

The convergence of widespread antibiotic resistance and the emergence of hypervirulent community-acquired strains has established *KP* as a critical public health threat. This urgency underscores the need for accelerated vaccine development capable of targeting both healthcare-associated and community-acquired infections, particularly those caused by hypervirulent variants [76]. Numerous vaccine candidates have been proposed, with several advancing to clinical trials. This section reviews the current landscape of *KP* vaccine development, covering strategies such as whole-cell vaccines, OMVs, polysaccharide- and protein-based formulations, and ribosomal vaccines. Special emphasis is placed on conjugate, nanomaterial-based, and mucosal vaccines (Table 1). Collectively, these efforts confirm that a *KP* vaccine is both feasible and promising for infection prevention and reducing the global burden of AMR.

**Table 1:** Vaccine candidates for *Klebsiella pneumoniae.*

Vaccine type		Year	Antigen	Animal	Immunized Route	Challenge method	Refs
Inactivated		2011	Heat-killed *KP*-43816 (ATCC) (serotype K2)	C57BL/6 mice	IN	Pulmonary	[77]
		2021	Heat-killed *KP*	BALB/c mice	ID	Systemic	[78]
		2022	Formaldehyde inactivated *hvKP*	CD1 mice	SQ	Systemic	[79]
Live attenuated		2008	*TonB* mutant *KP*	BALB/cByl mice	IP	Systemic	[80]
		2014	A *sitA* mutant of *KP*	BALB/c mice	IP	Systemic	[81]
		2022	*KbvR* knockout in *hvKP*	BALB/c mice	SQ	Systemic	[82]
		2022	*MurI* knockout bacteria	BALB/c mice	IP	Systemic	[83]
Polysaccharide		2008	Purified LPS antigen	BALB/c mice	IP	Systemic	[84]
		2014	A LPS D-galactan II-producing strain	BALB/cByl mice	IP	Systemic	[85]
		2018	CPS2	New Zealand white rabbits	IM	N/A	[86]
		2019	CPS2	Cynomolgus macaques	IM/SQ	Pulmonary	[87]
Glyco-conjugate	Chemical conjugate	2017	Polysaccharide conjugated to CRM197-1	Mice/Rabbits	SQ	N/A	[88]
		2018	OPS conjugated to flagellin *(*FlaA, FlaB)	Crl:CD-1 mice	IM	Systemic	[36]
		2020	K2 CPS conjugated to diphtheria toxoid	BALB/c mice	IM/SQ	N/A	[89]
		2022	K1 and K2 capsule oligosaccharides conjugated to CRM197	Unspecified mice	IM	Systemic	[90]
		2023	Outer core oligosaccharides from LPS conjugated to CRM197 or BSA	C57BL/6J mice	IM	N/A	[91]
		2024	O2agf antigens conjugated to CRM197	Rabbits	IM	Pulmonary	[92]
	Biosynthetic glycoconjugate	2019	K1 and K2 CPS conjugated to EPA	BALB/c mice	SQ	Pulmonary	[93]
		2023	K1 CPS and O1 O-antigen conjugated to EPA	BALB/c mice	SQ	Pulmonary/systemic	[94]
		2024	O-antigen conjugated to EPA	CD-1 mice	N/A	N/A	[95]
		2025	K2 CPS and O1 O-antigen conjugated to EPA	CD-1 mice	SQ	Systemic	[96]
Protein	OMP	2018	OmpK17, OmpK36 and the fusion protein F36/17	Female Swiss albino mice	SQ	Systemic	[97]
		2021	OMPs (Kpn_Omp001/002/003)	BALB/c mice	SQ	Pulmonary/Systemic	[98]
		2022	OmpK36 and OmpC	BALB/c mice	SQ	Systemic	[99]
		2022	PhoE	BALB/c mice	SQ	Systemic	[100]
	Fimbria	2005	Type 3 fimbriae	BALB/c mice	IP	Pulmonary/Systemic	[101]
		2024	FimA/FimC/FimG	BALB/c mice	SQ	Systemic	[102]
		2025	FimA and MrkA	C57BL/6 mice	SQ	Pulmonary	[103]
	Multivalent protein	2010	Epitopes within the MrkD antigen (M221-235, M175-189, and M264-278)	BALB/c mice	SQ	N/A	[104]
		2017	OmpA and OmpK36	BALB/c mice	SQ	Systemic	[105]
		2024	FepA, OmpA, OmpW, OmpK36 and OmpK17	BALB/c mice	IM	Pulmonary	[106]
	Siderophore Receptor proteins	2018	Siderophore Receptor proteins	Cows	IM	N/A	[107]
Nanovaccines	OMV	2015	Bacteria-derived extracellular vesicles	C57BL/6 mice	IP	Systemic	[108]
		2020	OMVs stabilized with BSA nanoparticles	C57BL/6 mice	SQ	Systemic	[109]
		2021	Bacterial biomimetic vesicles	ICR mice and C57BL/6N mice	SQ	Pulmonary/systemic	[110]
	Polysaccharide	2021	O2 O-antigen conjugated to cholera toxin B as nanoconjugate	BALB/c mice	SQ	Pulmonary/systemic	[111]
		2023	O1/O2 OPS assembled on a nanoparticle via SpyTag/SpyCatcher	BALB/c mice	SQ	Systemic	[112]
		2024	OPS-conjugated, self-assembled cholera toxin B subunit nanoparticle (CNP-OPS)	BALB/c mice	SQ	Systemic	[113]
		2025	OPS conjugated to hepatitis B virus core antigen (HBc)	BALB/c (SPF) mice	IM	Systemic	[114]
	OMP	2025	PhoE encapsulated in zeolitic imidazolate framework-8 (ZIF‐8)	BALB/c mice	SQ	Systemic	[115]
Mucosal	Polysaccharide	2015	LPS loaded alginate microparticles	New Zealand white rabbits/Swiss albino mice	IN/IM	Pulmonary	[116]
		2023	K2O1 capsular antigen in MPEG-PCL/PLGA nanoparticles	Unspecified mice	IN	Pulmonary	[117]
	OMP	2020	FyuA	BALB/c mice	IN	Pulmonary	[118]
		2021	Omp X and LTA1 adjuvant	WT C57BL/6J mice	IT	Pulmonary	[119]
		2023	rOmpA encapsulated in silk fibroin-sodium alginate nanoparticles	BALB/c mice	IN	Pulmonary	[120]
		2024	OmpA	BALB/c mice	IN	N/A	[121]
		2025	GlnH and FimA	C57BL/6 mice	IN	Pulmonary	[122]
	OMV	2024	Biomimetic hybrid membrane vesicles (HMVs)	BALB/c mice	IT	Pulmonary	[123]
Ribosomal		1980	Ribosomal proteins	BALB/c mice	SQ	Systemic	[124]
		1982	Ribosomal proteins	BALB/c mice	SQ	Systemic	[125]
Nucleic acid vaccine		2010	OmpA and OmpK36	BALB/c mice	ID/IM	Systemic	[126]
		2024	YidR (IL-17 cytokine as adjuvant)	Kunming (KM) mice	IM	Pulmonary	[127]

Abbreviations: *KP*, *Klebsiella pneumoniae*; *hvKP*, hypervirulent *Klebsiella pneumoniae*; LPS, Lipopolysaccharide; CPS, Capsular Polysaccharide; CRM197, Cross-Reactive Material 197; OPS, O-polysaccharide; BSA, Bovine Serum Albumin; EPA, *Pseudomonas* exotoxin A; OMP, Outer Membrane Protein; OMV, Outer Membrane Vesicle. ID, intradermal; IM, intramuscular; IN, intranasal; IP, intraperitoneal; N/A, not available; SQ, subcutaneous; IT, Intratracheal.

#### 4.3.1 Whole-cell vaccines

##### 4.3.1.1 Inactivated vaccines

Inactivated *KP* vaccines are produced through heat or chemical treatment (e.g., formaldehyde fixation), which abolishes bacterial viability while preserving immunogenic epitopes. These vaccines benefit from a short development cycle and the elimination of pathogenicity while retaining immunogenicity.

Early inactivated *KP* vaccines were primarily developed for veterinary use, often formulated as multivalent combinations. Recent studies, however, support their potential in human applications. Immunization with heat-killed *KP* has been shown to elicit a robust Th17-mediated immune response and confer significant protection in murine pneumonia models [77]. Intradermal administration of heat-killed MDR-*KP* significantly improved survival rates in mice challenged with MDR-*KP* [78]. Another study using subcutaneous delivery revealed that the bacterial capsule promotes bloodstream survival by evading phagocytosis by liver Kupffer cells (KCs)—a key virulence mechanism that can be counteracted through vaccination [79].

Overall, inactivated whole-cell vaccines have demonstrated efficacy against preventing respiratory and urinary tract *KP* infections. However, toxicity concerns and incomplete validation of the duration of protection and precise immune mechanisms remain limitations to their broad application.

##### 4.3.1.2 Live-attenuated vaccines

Live-attenuated vaccines are developed by reducing bacterial virulence while preserving their ability to elicit protective immunity. They typically induce stronger and more durable immune responses than subunit vaccines, often at lower production costs [80,82]. Nevertheless, potential safety issues, such as reversion to virulence, restrict their use in immunocompromised populations, limiting current application to healthy individuals or livestock.

Genetic engineering has markedly improved the safety profiles of modern attenuated vaccines. For example, intraperitoneal administration of *KP tonB* deletion mutants provided cross-protective immunity against wild-type strains in mouse models [80]. Vaccination with inactivated *ΔSitA* mutants conferred better protection against wild-type challenge than either inactivated wild-type or capsule-deficient variants [81]. A promising glutamate racemase (*MurI*)-deficient mutant vaccine delivered intravenously induced robust immunity and cross-protection against multiple *KP* strains, including MDR isolates [83].

Notably, the *ΔkbvR* mutant has emerged as a highly promising vaccine candidate due to its attenuation profile. Subcutaneous immunization with this strain elicited strong humoral responses, enhanced OPK and complement-mediated bacteriolysis, and provided cross-serotype protection against heterologous challenges [82]. These findings underscore the considerable translational potential of live-attenuated vaccines for preventing *KP* infections in humans.

#### 4.3.2 Subunit vaccines and recombinant vaccines

##### 4.3.2.1 Polysaccharide vaccines

###### 4.3.2.1.1 Capsular polysaccharide vaccine

CPS is a key target in *KP* vaccine development due to its immunogenicity. However, the extensive serotype diversity of *KP*—with over 100 identified to date—poses a major challenge [31]. Given the marked differences in virulence among serotypes, research has prioritized hypervirulent types such as K1 and K2, aiming to leverage their dominant antigenic profiles for vaccine development.

Kobayashi et al. suggested that enhancing neutrophil phagocytosis could serve as a therapeutic strategy against ST258 CR-*KP* infections [128]. Follow-up studies demonstrated that rabbit immune serum and purified IgG targeting ST258 CPS2 significantly increased serum bactericidal activity in human blood and serum *in vitro*. Furthermore, CPS2-specific antibodies strongly enhanced opsonophagocytosis and killing of ST258 strains by human neutrophils [86]. Malachowa et al. established a novel ST258 pneumonia model in cynomolgus macaques and observed that all animals immunized with CPS2 produced ST258-specific antibodies. These antibodies substantially improved serum bactericidal activity and neutrophil-mediated killing *in ex vivo* assays [87]. Collectively, evidence from rodent to primate models strongly supports CPS as a promising antigen for *KP* vaccine development.

###### 4.3.2.1.2 Lipopolysaccharide vaccine

In contrast to the highly diverse CPS (over 100 serotypes), LPS contains only 11 known O-antigen serotypes, offering a more streamlined target for vaccine design. Notably, four serotypes—O1, O2, O3, and O5—account for over 90% of clinical isolates, suggesting that multivalent O-antigen or O1-specific vaccines could have broad clinical utility [129].

Immunization with purified LPS has been shown to confer protection against lethal *KP* challenge in mice [84]. Hsieh et al. further demonstrated that D-galactose-II is involved in LPS biosynthesis, and immunization with a strain producing LPS D-galactan-II protected against infection by an O1: K2 liver abscess strain [85]. LPS offers several advantages as a vaccine target: it is surface-exposed and has limited serotypic variation. To maximize its potential, current strategies focus on improving safety through detoxification (via genetic or chemical methods) and enhancing immunogenicity with novel adjuvants. Particularly promising are polysaccharide-protein conjugate vaccines, which represent a leading direction for future LPS-based vaccine development.

##### 4.3.2.2 Protein vaccines

###### 4.3.2.2.1 Outer membrane protein vaccines

OMPs of *KP* have garnered significant attention as promising vaccine targets due to their roles in host-pathogen interactions and their ability to induce protective immunity. OmpA, for instance, binds to bronchial epithelial cells and activates the NF-κB pathway, enhancing chemokine production and modulating neutrophil recruitment to the lungs [130]. Immunization with OmpK17, OmpK36, or a recombinant fusion protein F36/17 significantly improved survival in murine models. Serum profiling indicated a Th2-biased response, characterized by elevated IgG1 over IgG2a levels [97]. Additionally, intraperitoneal administration of *KP* OmpK36 and *Salmonella Typhi* OmpC elicited specific antibody responses against both antigens in BALB/c mice [99].

**Fig. 3:**
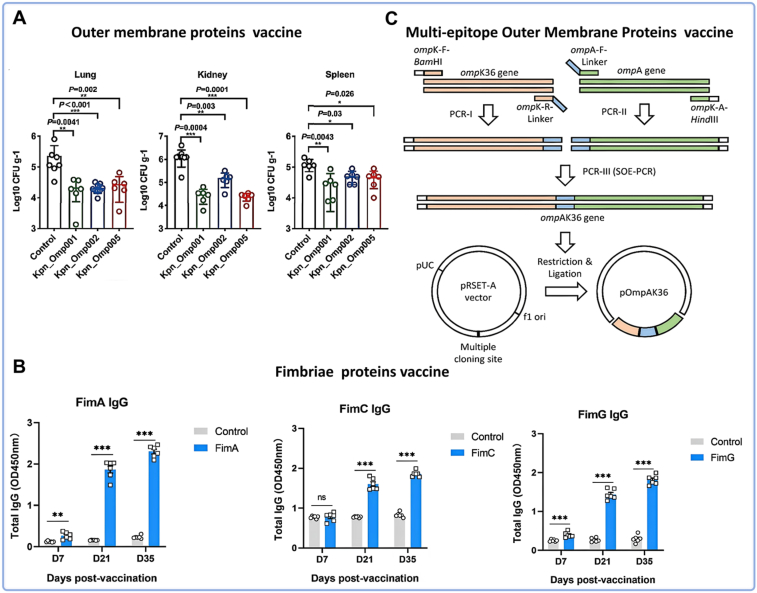
Schematic representation of protein vaccine types and their therapeutic efficacy against *KP* infection. (A) Bacterial burdens in the lungs, kidney, and spleen were calculated after infection [98]. (B) Antibody titer levels induced by fimbrial protein vaccines [102]. (C) Gene editing process for multi-epitope outer membrane protein vaccine design [105].

Advances in bioinformatics and proteomics have enabled more rational vaccine design. Computational screening identified three highly antigenic OMPs—KPn_Omp001, KPn_Omp002, and KPn_Omp005. Subcutaneous immunization with these recombinant proteins induced robust humoral (IgG, IgG1, IgG2a) and cellular (IFN-γ, IL-4, IL-17a) immune responses, conferring protection against both pneumonia and bacteremia in mice (Fig. 3A) [98]. Similarly, PhoE, an efflux channel associated with antibiotic resistance, elicited strong humoral and cellular immunity following subcutaneous vaccination in mice [100]. These studies exemplify a shift from empirical antigen screening toward structure-informed, rational antigen selection, offering novel strategies to counter drug-resistant *KP*.

###### 4.3.2.2.2 Fimbrial protein vaccines

Fimbriae are key virulence factors facilitating biofilm formation and host cell adhesion. Type I and type III fimbriae—especially the Mrk adhesins—are attractive vaccine targets due to their conservation and surface accessibility. Subcutaneous immunization with recombinant FimA and MrkA proteins induced high IgG1 titers in mice [103]. Similarly, intraperitoneal delivery of three fimbrial proteins (FimG, FimA, FimC) elicited robust humoral responses, with significant increases in IgG, IgG1, and IgG2a compared to controls (Fig. 3B) [102]. Lavender et al. further demonstrated that intraperitoneal administration of purified type III fimbriae significantly improved survival in *KP*-challenged mice [101]. These findings underscore the potential of fimbrial proteins as effective vaccine antigens.

###### 4.3.2.2.3 Multi-epitope vaccines

Multi-epitope vaccines represent a cutting-edge approach enabling precise immune targeting and broad-spectrum coverage. In *KP* research, bioinformatics-driven epitope prediction has identified immunogenic regions in OMPs such as OmpA, OmpK36, FepA, OmpK17, and OmpW [131]. A recombinant multi-epitope protein, r-AK36, containing domains from OmpA and OmpK36, was developed as a subunit vaccine. Immunization with r-AK36 stimulated protective cytokines (IL-2 and IFN-γ) and induced a Th2-polarized humoral response dominated by IgG1 (Fig. 3C) [105]. Liao et al. developed mHla-EpiVac, a multi-antigen vaccine incorporating five OMPs (OmpA, OmpK36, FepA, OmpK17, OmpW), which elicited strong humoral and cellular immunity upon intramuscular delivery in mice [106]. Fimbrial proteins have also been exploited; three MrkD-derived Th-cell epitopes (M221–235, M175–189, M264–278) effectively activated antigen-specific CD4^+^ T-cell responses [104].

These efforts highlight the feasibility of multi-epitope vaccines for *KP* and provide a rational framework for developing broad-spectrum formulations. By overcoming the limitations of single-antigen vaccines, multi-epitope designs induce synergistic immune responses for comprehensive protection.

###### 4.3.2.2.4 Siderophore receptor protein vaccines

Siderophore receptors, essential for iron acquisition and virulence, are emerging as viable vaccine targets. A veterinary vaccine (Kleb-SRP) based on SRPs reduced the incidence of *KP*-associated bovine mastitis and increased milk yield, while also conferring cross-protection against *Escherichia coli* (*E. coli*) infections [107]. In addition, a comprehensive computational study systematically identified T-cell (class I and II) and B-cell (linear and conformational) epitopes within FepA, enabling a transition from whole-protein vaccines to precision epitope-based design [132]. These advances deepen our understanding of *KP* pathogenesis and support the development of multi-targeted vaccines for preventing and controlling *KP* infections.

##### 4.3.2.3 Glycoconjugate vaccines

Glycoconjugate vaccines represent an advanced class of immunogens created by covalently linking bacterial polysaccharides to protein carriers. This strategy converts thymus-independent (TI) polysaccharide antigens into thymus-dependent (TD) antigens, thereby overcoming the limitations of pure polysaccharide vaccines, which elicit weak and short-lived T cell-independent responses. Glycoconjugate vaccines induce robust IgG production even in immunologically vulnerable populations such as infants, the elderly, and immunocompromised individuals [133]. Current manufacturing strategies primarily include chemical conjugation and biosynthetic platforms.

###### 4.3.2.3.1 Chemical preparation of conjugate vaccines

**Fig. 4:**
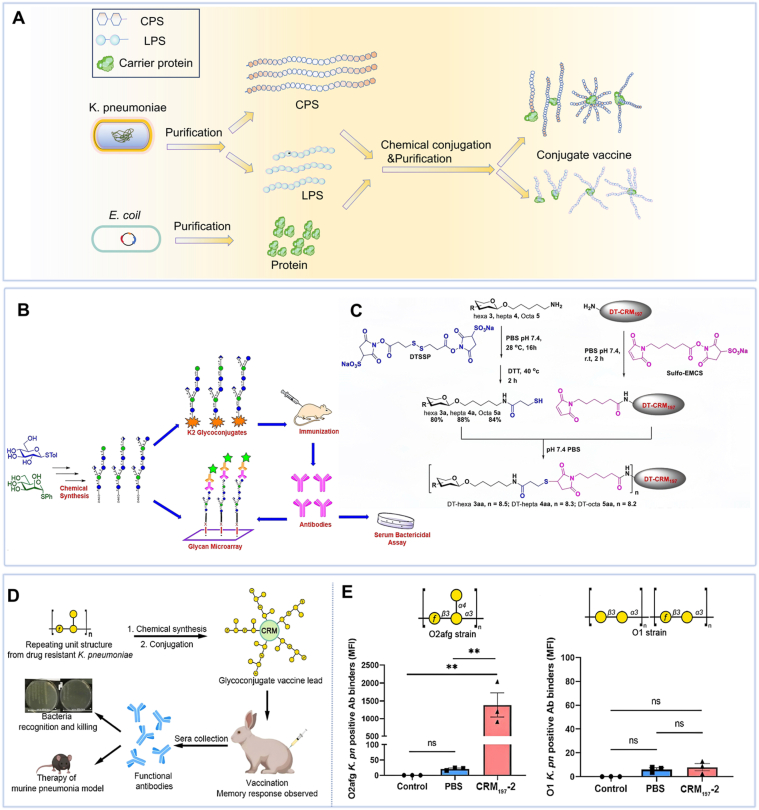
Chemically conjugated vaccines. (A) Schematic of the chemical conjugation vaccine preparation process. Created with Biorender.com. (B-C) Schematic and molecular structure of a K2 CPS-conjugated [89]. (D) A schematic diagram illustrating the conjugated vaccine-induced O2afg serotype-specific antibodies and their protective effect against *KP* infection. (E) Rabbit-derived antisera were assessed for opsonic killing activity against *KP* O2afg and O1 strains *in vitro* and *in vivo* [92].

Chemical conjugation is a well-established method for producing polysaccharide-protein conjugate vaccines. The process involves four key steps: (1) isolation and purification of polysaccharides and carrier proteins; (2) chemical activation to introduce reactive groups; (3) controlled crosslinking to form stable covalent bonds; and (4) comprehensive purification to obtain high-purity conjugates (Fig. 4A) [134,135]. The use of well-defined, low-molecular-weight oligosaccharides is often preferred to ensure batch consistency and enable precise correlation between structure and immune response [136,137].

CPS-based conjugate vaccines have been extensively studied. K1 and K2 oligosaccharides conjugated to cross-reactive material 197 (CRM197) carrier protein elicited potent anti-CPS antibodies and enhanced serum bactericidal activity in mice [90]. Ravinder et al. synthesized three glycoconjugates by linking K2 CPS to diphtheria toxoid, all of which induced functional antibodies with significant bactericidal activity (Fig. 4B and C) [89]. Seeberger et al. developed a semi-synthetic hexasaccharide conjugate that elicited cross-reactive antibodies against CR-*KP* CPS in both mice and rabbits, demonstrating protective efficacy in challenge models [88].

Approximately 80% of clinical *KP* isolates belong to four O-serotypes (O1, O2, O3, O5), making O-antigens ideal targets for glycoconjugate vaccines [138]. Hegerle et al. developed a multivalent glycoconjugate vaccine incorporating O1, O2, O3, and O5 polysaccharides conjugated to (*Pseudomonas aeruginosa*) *PA* flagellins (FlaA, FlaB). This formulation induced robust antibody responses against all six antigens in rabbits, with flagellin conjugation notably enhancing anti-polysaccharide immunogenicity [36]. Chen et al. chemically synthesized tetra- and pentasaccharides derived from *KP* LPS outer core structures, conjugated them to CRM197 and Bovine Serum Albumin, and demonstrated that these glycoconjugates elicited antibodies recognizing purified LPS and multiple *KP* strains [91]. For the prevalent CR-*KP* serotype O2afg (ST258), a semisynthetic glycoconjugate vaccine conjugated to CRM197 elicited functional IgG antibodies in rabbits that mediated OPK and enhanced complement activation (Fig. 4D–E) [92].

Glycoconjugate vaccines currently represent the most promising approach for *KP* vaccination, demonstrating substantial preclinical efficacy through enhanced immunogenicity and protective immunity. However, clinical translation faces challenges including complex manufacturing, potential antigen degradation during chemical conjugation, batch-to-batch variability, inconsistent immunogenicity, and high production costs. These limitations highlight the need for next-generation platforms that improve manufacturing efficiency and product consistency.

###### 4.3.2.3.2 Biological preparation of glycoconjugate vaccines

**Fig. 5:**
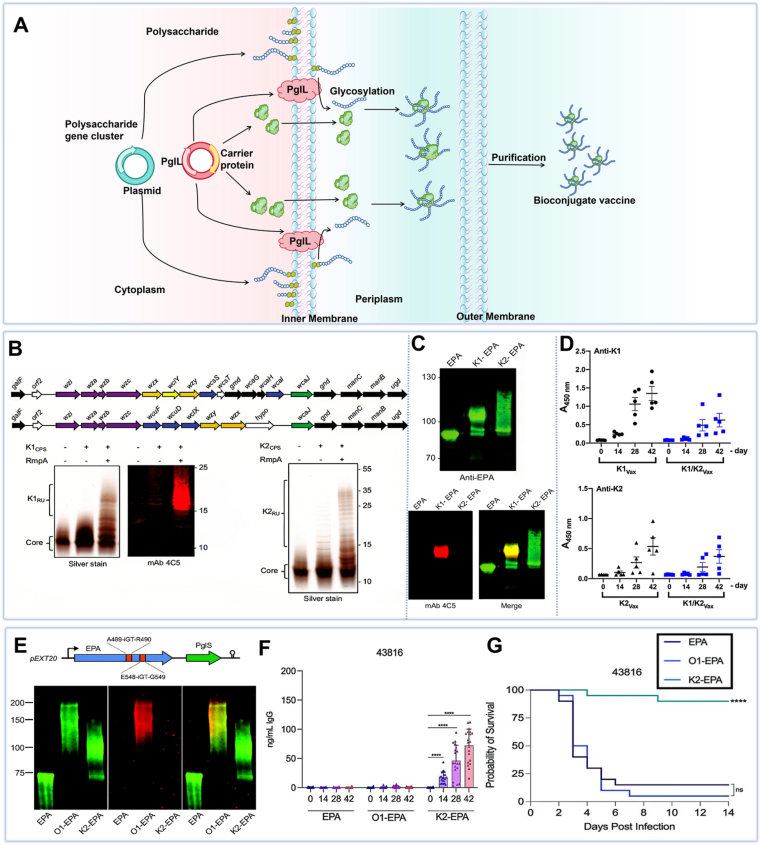
Biosynthetic glycoconjugate vaccines. (A) Schematic of the biosynthetic glycoconjugate vaccine production process. Created with Biorender.com. (B) Genetic design and Western blot analysis of K1-EPA/K2-EPA biosynthetic glycoconjugate vaccines. (C) Western blot detection of EPA and K1 polysaccharide expression. (D) IgG antibody levels induced by K1- and K2-EPA biosynthetic glycoconjugate vaccines [93]. (E) Characterization of O1-EPA and K2-EPA biosynthetic glycoconjugates. (F) Vaccine-induced IgG antibody production. (G) Vaccine protective efficacy on survival rates [94].

Biosynthetic glycoconjugate vaccines are produced either in native host systems or in glycoengineered *E. coli* strains, enabling simultaneous expression of glycan antigens and carrier proteins followed by efficient *in vivo* conjugation. In this process, the polysaccharide is synthesized on an undecaprenyl pyrophosphate lipid anchor in the cytoplasm and subsequently translocated to the periplasm. There, an oligosaccharyltransferase recognizes the lipid-linked glycan at its reducing end and catalyzes its transfer to a conserved acceptor sequon within the carrier protein, forming the final biosynthetic glycoconjugate (Fig. 5A) [139,140]. This approach offers significant advantages by enabling coordinated expression and coupling of both components *in vivo*, substantially simplifying production and improving consistency compared to chemical methods [133].

Biosynthetic glycoconjugate vaccines are generated through two primary pathways: homologous and heterologous expression systems. In homologous expression, an O2 serotype polysaccharide conjugate vaccine produced using *KP*'s endogenous system elicited robust antibody responses and significantly improved survival rates in mice following intraperitoneal immunization [141]. Heterologous expression systems, however, demonstrate greater versatility and control. By introducing plasmids containing target polysaccharide genes, carrier proteins, and glycosyltransferase enzymes into *E. coli*, the endogenous bacterial glycosylation machinery can be harnessed to express and conjugate the desired polysaccharide to the carrier protein [133]. For instance, glycoengineered *E. coli* strains have been developed to produce a bivalent K1/K2 *KP* biosynthetic glycoconjugate vaccine (Fig. 5B and C). These biosynthetic glycoconjugates demonstrated strong immunogenicity and provided complete protection in mice challenged with hypervirulent strains NTUH-K2044 and ATCC 43816 (Fig. 5D) [93]. A subsequent study expanded this platform to develop a novel bivalent vaccine simultaneously targeting K2 CPS and O1 O-antigen (Fig. 5E and F). Subcutaneous immunization significantly enhanced survival in murine challenge models (Fig. 5G) [94]. Furthermore, Wantuch et al. evaluated the long-term immunogenicity of biosynthetic glycoconjugate vaccines targeting serotype K2 and O1v1 O-antigen, confirming sustained IgG titers for at least six months post-immunization in mice [96].

Biosynthetic glycoconjugation offers substantial technical advantages over traditional chemical methods, effectively addressing issues such as product heterogeneity and immunogenic inconsistency. Heterologous expression systems utilizing *E. coli* have been particularly transformative, enabling precise regulation of polysaccharide expression and chain length. This system leverages glycosyltransferases for site-specific conjugation to carrier proteins, significantly enhancing vaccine quality [139,142]. Additionally, *E. coli'*s rapid growth, ease of cultivation, and compatibility with diverse polysaccharides and carrier proteins make it highly suitable for large-scale production. Numerous studies indicate that biosynthetic glycoconjugation of *KP* LPS to carrier proteins represents a highly promising strategy, with strong potential for clinical translation. Wantuch et al. developed a multivalent biosynthetic glycoconjugate vaccine targeting seven predominant *KP* O-antigen subtypes. Each component elicited robust immunogenicity individually, and vaccine-induced antibodies exhibited broad cross-reactivity against diverse *KP* strains while mediating complement-dependent killing, demonstrating functional potency [143].

##### 4.3.2.4 Nanovaccines

Nanovaccines represent an advanced class of biologics that utilize nanomaterials as carriers, linkers, or immunomodulators. They are combined with target antigens and adjuvants through physical or chemical methods to enhance preventive or therapeutic efficacy. Compared to soluble antigens, nanoparticle formulations significantly improve antigen cross-presentation and CD8^+^ T cell activation [144]. They enhance payload bioavailability through improved tissue penetration and targeted delivery to immune cells [145], as well as enable sustained release to maintain antigen immunogenicity over extended periods [146]. Additionally, their size-dependent properties promote efficient trafficking to lymphoid organs and enhance uptake by antigen-presenting cells, further amplifying immune responses [[146], [147], [148]]. A variety of nanomaterials—including liposomes [109,110], polymeric nanoparticles [117,149], and inorganic nanoparticles [115,150]—have been widely employed in vaccine development.

###### 4.3.2.4.1 Outer membrane vesicle nanovaccines

OMVs are lipid bilayer nanoparticles (50–250 nm) that naturally drain to lymph nodes, facilitating direct recognition by B cells and induction of potent humoral immunity. They carry a broad repertoire of immunostimulatory antigens, overcoming the limited immunogenicity of single-antigen formulations. As non-replicating and metabolically inert structures, OMVs furthermore avoid the safety risks associated with live vaccines. Lee et al. demonstrated that extracellular vesicles derived from *KP* confer significant protection against lethal bacterial challenge [108]. The integration of OMVs with other nanomaterials further reduces potential toxicity while enhancing immunogenicity, particularly against the drug-resistant *KP* [109]. These collective advantages position OMVs as a highly promising platform for next-generation nanovaccines.

**Fig. 6:**
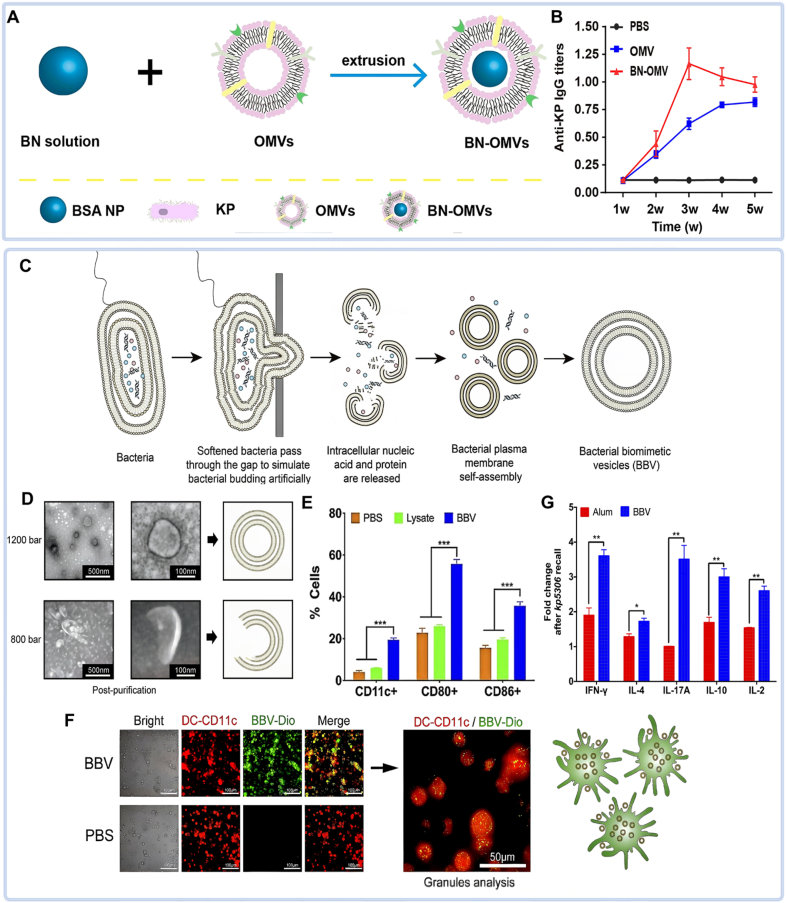
OMV-based nanovaccines. (A) Preparation process of BN-OMV nanovaccine. (B) IgG antibody titers induced by BN-OMV vaccination [109]. (C) Design of BBVs nanovaccine. (D) TEM images of vaccine structure. (E) Analysis of cellular immunity. (F) Dendritic cell uptake demonstrated by co-localization staining. (G) Inflammatory cytokine profiles [110]. Abbreviation: BN-OMV, the hybrid nanoparticle formed by depositing hollow outer membrane vesicles onto bovine serum albumin nanoparticles; BBVs, bacterial biomimetic vesicles.

Wu et al. developed structurally reinforced OMVs by encapsulating size-controlled Bovine Serum Albumin nanoparticles within hollow OMVs via hydrophobic interactions (BN-OMVs) (Fig. 6A). Subcutaneous administration of these BN-OMVs elicited substantially higher antigen-specific antibody titers against CR-*KP* and significantly improved survival in mice challenged with lethal doses of CR-*KP* (Fig. 6B) [109]. In another innovative approach, high-pressure extrusion was used to force *KP* through microapertures, inducing the formation of self-assembling bacterial biomimetic vesicles (BBVs) (Fig. 6C and D). These BBVs were efficiently internalized by dendritic cells (DCs), promoting their maturation. As a vaccine platform, BBVs elicited dual protective immunity—inducing both humoral and cellular responses—which significantly enhanced survival, reduced pulmonary inflammation, and decreased bacterial burden in challenged animals (Fig. 6E–G) [110]. These advances underscore the strong clinical potential of OMV-based nanovaccines for combating *KP* infections.

###### 4.3.2.4.2 Nano-glycoconjugate vaccines

Biosynthetic technologies have enabled a transformative shift from traditional glycoconjugate vaccines to advanced nano-glycoconjugate vaccines, significantly enhancing immunogenicity and structural homogeneity. Current biosynthetic strategies primarily utilize two expression systems: natural host expression and engineered heterologous expression in *E. coli*. A representative approach employs the SpyTag/SpyCatcher covalent conjugation platform, in which the SpyCatcher domain is genetically fused to O1 serotype polysaccharide antigens. These are then assembled onto SpyTag-functionalized AP205 virus-like particles (VLPs), yielding structurally precise KPO1-VLPs and KPO2-VLPs. This modular system provides a versatile and scalable platform for developing biosynthetic *KP* nanovaccines with controlled antigen display [151].

Owing to its superior capabilities in heterologous expression, *E. coli* has become the preferred system for polysaccharide nanovaccine production, offering advantages in industrial scalability and product consistency. For instance, an engineered *E. coli* chassis was developed to produce glycoproteins decorated with antigenic polysaccharides. Through SpyCatcher/SpyTag-mediated *in vitro* conjugation, these glycoproteins were assembled into well-defined nanovaccines using protein nanoparticle carriers. The resulting O1 conjugate nanovaccine (*KP*O1-VLP) induced high-titer, antigen-specific antibodies after triple immunization and conferred complete protection against lethal challenge with virulent *KP* strains [112]. Similarly, Zhang et al. constructed an *E. coli* platform to produce self-assembling nanoparticles based on the cholera toxin B subunit (CTB), which were covalently linked to *KP* O-polysaccharide (OPS) to form a biosynthetic glycoconjugate nanovaccine (CNP-OPS). Subcutaneous immunization with CNP-OPS elicited robust O2-specific immune responses and demonstrated significant protective efficacy in murine infection models [113].

**Fig. 7:**
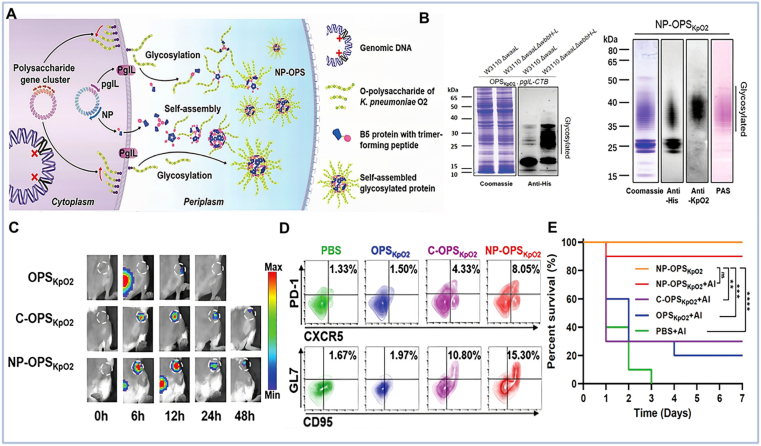
Nano-glycoconjugate vaccines. (A) Schematic illustration of the nano-glycoconjugate vaccines. (B) Validation of *KP* O2 polysaccharide expression and conjugate formation using Western blot and SDS-PAGE. (C) Drainage kinetics of fluorescently labeled vaccine formulations to the draining lymph nodes over time. (D) Analysis of Tfh cell populations (CXCR5^+^PD-1^+^ within CD4^+^ cells) and germinal center B cells (GL7^+^CD95^+^ within B220^+^ cells) in lymphoid follicles. (E) Evaluation of vaccine-mediated protection efficacy on survival rates [111].

The O2 serotype of AMR-*KP*, responsible for 35–59% of clinical infections, poses a serious public health threat, underscoring the urgent need for effective vaccines [114]. Although *KP* O2 OPS is an attractive antigen candidate, its low immunogenicity has limited clinical application [111,114]. To overcome this, Peng et al. developed a versatile *E. coli*-based platform using the nano-B5 self-assembly system to produce nanoscale conjugate vaccines against *KP* (Fig. 7A and B). Their nanoconjugate vaccine induced efficient humoral immune responses in draining lymph nodes and elicited high titers of anti-LPS IgG antibodies (Fig. 7C–E) [111]. In an alternative innovative strategy, hepatitis B core antigen (HBc) nanoparticles were utilized as carriers for OPS presentation. By integrating protein-glycan coupling technology (PGCT) with the SpyCatcher/SpyTag system, an HBc-OPS biosynthetic glycoconjugate vaccine was developed. Murine studies demonstrated that this vaccine could elicit OPS-specific antibodies and provide protection against a broad range of infectious doses [114].

The biosynthetic glycoconjugation approach enables precise attachment of polysaccharides to nanoparticle carriers. This strategy preserves antigen integrity and leverages nanoscale properties for enhanced delivery and presentation, which significantly potentiates immunogenicity. Successful validation in murine models of *KP* infection underscores its promise as an effective prophylactic intervention against *KP*.

###### 4.3.2.4.3 Protein nanovaccines

OMPs such as OmpK17, OmpK36, and PhoE have demonstrated high accessibility and immunogenicity, making them attractive targets for vaccine development [100]. Hu et al. developed a novel nanovaccine platform using zeolitic imidazolate framework-8 (ZIF-8) to encapsulate PhoE antigen. The ZIF-8 nanoparticles facilitated efficient delivery of PhoE into DCs, leading to successful cellular activation. Mice subcutaneously immunized with PhoE@ZIF-8 exhibited significantly higher IgG antibody titers, elevated cytokine levels, and increased splenocyte proliferation compared to controls [115].

#### 4.3.3 Ribosomal vaccines

Ribosomal vaccines, composed of purified bacterial ribosomes or ribosomal subunits, have shown superior protective efficacy compared to whole-cell vaccines [152]. Animal studies confirmed their effectiveness, showing that immunization with purified ribosomes or ribosomal proteins significantly reduced pathology in mice infected with homologous *KP* strains [124]. These findings were further supported by mechanistic studies: adoptive transfer of serum and splenocytes from mice immunized with rRNA plus adjuvant protein conferred protection primarily via cell-mediated immunity [125]. Moreover, ribosomes possess dual immunomodulatory capacity: they activate T-cell responses while also enhancing B-cell proliferation and antibody production [153].

However, subsequent studies attributed the observed immunogenicity not to ribosomes per se, but to residual bacterial components—including rRNA, proteins, and LPS—that persist through purification. This recognition has led to the gradual discontinuation of ribosomal vaccines in favor of more specific and controllable antigen platforms.

#### 4.3.4 Nucleic acid vaccines

Nucleic acid vaccines represent an innovative approach that delivers mRNA or DNA sequences encoding target antigens, leveraging the host's cellular machinery for antigen production and subsequent immune activation. These vaccines offer three key advantages over conventional platforms: cost-efficient manufacturing, rapid production timelines, and the ability to tailor immune responses through precise molecular design and advanced delivery systems [154]. DNA vaccines specifically provide exceptional stability, enhanced safety profiles, flexible design capabilities, and the capacity to elicit robust immune responses [154]. Compared to DNA vaccines, mRNA platforms offer additional advantages in simultaneously encoding multiple antigens, enabling protection against diverse pathogens and variants in a single formulation [138].

Kurupati et al. developed a DNA vaccine by cloning genes encoding OmpA and OmpK36 into the pVAX1 plasmid vector. Immunoblot analysis confirmed that antibodies from immunized mice specifically recognized both OMPs. Additionally, mice vaccinated with these constructs showed elevated production of IL-12 and IL-10 [126]. Similarly, Lv et al. created a dual-plasmid system consisting of pVAX1-YidR (encoding the conserved virulence factor YidR) and pVAX1-IL-17 (expressing IL-17 as a molecular adjuvant). Co-immunization with both plasmids significantly enhanced adaptive immune responses and provided superior protection against *KP* challenge compared to single-component vaccination [127].

#### 4.3.5 Mucosal vaccines

Mucosal tissues form the primary barrier against external pathogens, lining the respiratory, gastrointestinal, and urogenital tracts. Although most licensed vaccines are administered via intramuscular or subcutaneous injection, these routes face limitations such as low patient compliance, risk of needle-related injuries, and inadequate elicitation of mucosal immunity. Aligning vaccine delivery with the natural routes of pathogen entry—particularly relevant for *KP*, which is primarily transmitted via the respiratory route—may offer more effective and targeted protection [155,156].

**Fig. 8:**
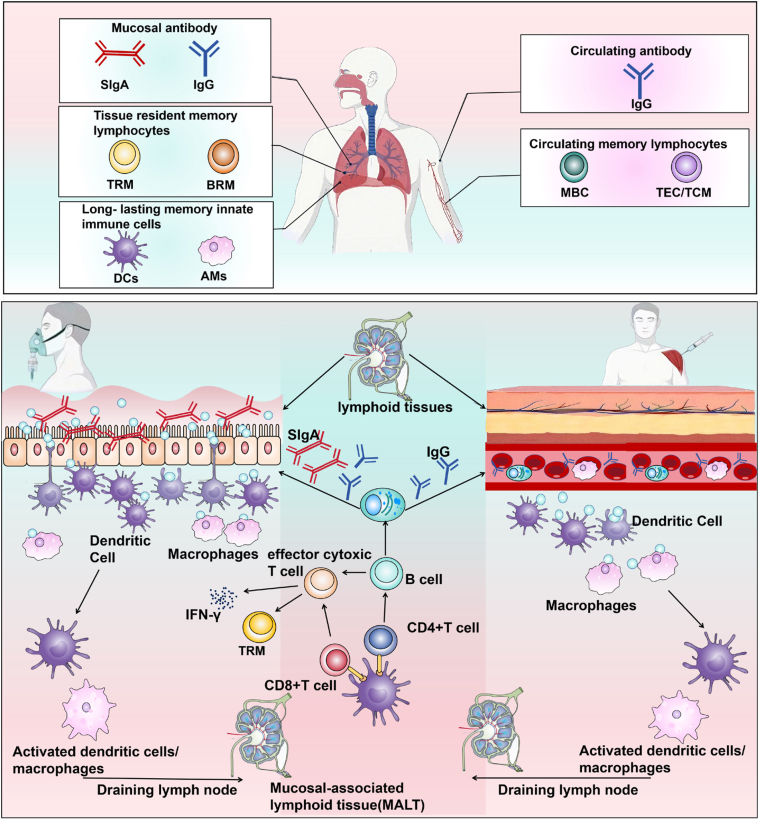
Comparison between mucosal vaccines and intramuscular vaccines. Created with Biorender.com. Abbreviations: TRM, Tissue-Resident Memory T cells; BRM, Tissue-Resident Memory B cells; IFN-γ, Interferon-γ; SIgA, Secretory Immunoglobulin A; MBC, Memory B Cell; TEC, Effector Memory T Cell; TCM, Central Memory T cells; AMs, Alveolar Macrophages.

Mucosal vaccination offers significant advantages by eliciting robust immune protection at primary pathogen entry and transmission. This approach effectively induces long-lasting B and T cell memory responses [157]. Antigen-primed B and T cells originating from mucosa-associated lymphoid tissue (MALT) migrate to effector sites, where they execute localized immune functions [158]. A key feature of mucosal immunity is the stimulation of antigen-specific SIgA production, which plays a critical role in immune exclusion by preventing pathogen adhesion and epithelial invasion [159]. Beyond SIgA, mucosal vaccination also activates CD4^+^ TRM T cells, triggering cytokine release and cytotoxic responses [160] (Fig. 8).

TRMs serve as pivotal cellular sentinels in this frontline defense. Following intranasal vaccination or infection, a subset of airway CD4^+^ and CD8^+^ T cells differentiates into CD69^+^CD103^+^ TRMs, forming stable, non-circulating populations at mucosal sites [161]. These cells exhibit unique phenotypic and transcriptional signatures, characterized by the expression of key surface markers (such as CD103, CD69, CD49a, and CXCR6) that are critical for their tissue-specific homing, retention, and function in peripheral sites like the lung, intestine, skin, and liver [162]. Their differentiation and maintenance are regulated by niche-specific cytokines: IL-13 from group 2 innate lymphoid cells (ILC2s) promotes TRM commitment, whereas IL-17 from ILC3s sustains their long-term persistence [162,163]. Importantly, intranasal or pulmonary inhalation vaccination not only induces TRM formation but also enhances germinal center reactions and fosters memory B cell development in the respiratory tract [163]. The synergistic interplay between TRM-driven local immunity and systemic immune memory ensures both immediate protection and durable surveillance, highlighting the rationale for next-generation vaccine platforms designed to efficiently generate TRMs.

Accumulating evidence underscores the capacity of mucosal immunization to elicit robust antibody responses at both local and systemic levels, underpinning its promise as a strategy for infectious disease control [15]. This section reviews the immunoenhancing effects of polysaccharide-, protein-, and OMV-based vaccines delivered via mucosal routes.

##### 4.3.5.1 Polysaccharide vaccines

Polysaccharide-based mucosal vaccines employ purified *KP* LPS or CPS encapsulated within inhalable nanoparticle delivery systems to enable efficient mucosal immunization. Studies indicate that such nanoparticles function as potent mucosal adjuvants, substantially enhance antigen uptake by antigen-presenting cells and boost overall immunogenicity. For instance, LPS encapsulated in alginate microparticles (∼5 μm) administered via intranasal or intratracheal routes effectively induced both systemic and mucosal immune responses, demonstrating suitability for large-scale vaccination programs with significant health and economic benefits [116]. Ghaderinia et al. developed Poly lactic-co-glycolic acid (PLGA) and Methoxypoly (ethylene glycol) Poly(caprolactone) (MPEG-PCL) nanoparticles encapsulating the capsular antigen of *KP* serotype K2 and O1. Mucosal administration of these nanoparticles induced robust IgA antibody responses in a murine model [117].

##### 4.3.5.2 Protein vaccines

Protein-based mucosal vaccines have shown considerable promise. One study developed a vaccine candidate by encapsulating recombinant OmpA (rOmpA) from *KP* in silk fibroin-sodium alginate nanoparticles (SF-SANPs). Intranasal administration significantly enhanced SIgA production and cytokine secretion, inducing both humoral (Th2) and cellular (Th1) immune responses, with a pronounced Th1 polarization [164]. Shahbazi et al. cloned the *ompA* gene into a pET-28a(+) vector and purified rOmpA. Intranasal immunization with rOmpA elicited significantly elevated levels of antigen-specific IgG antibodies against both rOmpA and whole *KP* cells compared to controls [121]. Beyond OMPs, SRPs such as FyuA have also been explored. Kumar et al. demonstrated that intranasal immunization with FyuA elicited robust humoral and cellular immune responses in mice, conferring significant protection against lethal *KP* challenge [118].

**Fig. 9:**
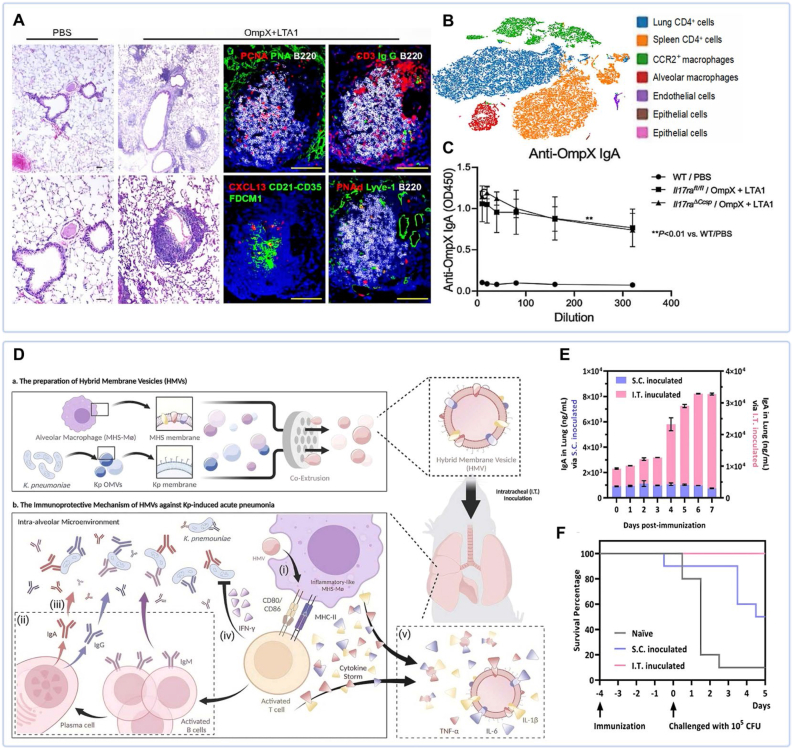
Mucosal vaccines. (A) H&E staining and multiplex immunofluorescence images of lung tissue architecture. (B) t-SNE plot of CD4^+^T cells from the spleens of naïve mice and the lungs of mice immunized with OmpX+LTA1 or heat-killed *KP*. (C) Comparative IgA antibody titers between unvaccinated and vaccinated groups [119]. (D) Schematic illustration of OMV-based mucosal vaccine preparation and inhalable vaccine delivery process. (E) Quantification of vaccine-induced IgA antibody titers. (F) Protective efficacy on survival rates [123].

TRM cells play a crucial role in pulmonary mucosal immunity. Recent studies show that mucosal vaccination can effectively induce TRM cells generation, establishing long-term local immune memory. Iwanaga et al. developed a vaccine comprising *KP* OmpX combined with the LTA1 adjuvant, delivered via intrapulmonary administration. This strategy effectively induced lung TRM cells and stimulated SIgA production in the pulmonary mucosa (Fig. 9A–C) [119]. Similarly, a bivalent subunit vaccine containing *KP* OMP (GlnH) and fimbrial adhesin FimA significantly improved survival rates in challenged animals. Critically, vaccine-induced TRM cells were identified in pulmonary tissues, suggesting their role in mediating protective immunity against bacterial pneumonia [122]. These findings underscore the therapeutic potential of TRM-eliciting vaccines for *KP* infections.

##### 4.3.5.3 Outer membrane vesicle vaccines

Bionanotechnology is advancing the development of OMV-based mucosal vaccines. By engineering physical properties (e.g., particle size and morphology) and biological characteristics (e.g., surface antigen density and distribution), bioinspired nanovaccines enhance antigen presentation efficiency and DC targeting specificity [123].

In a recent study, researchers developed biomimetic hybrid membrane vesicles (HMVs) by fusing *KP* OMVs with membrane vesicles derived from alveolar macrophages (AMs) (Fig. 9D). Intratracheal delivery of these HMVs induced robust pulmonary-specific immunity, characterized by elevated IgA and IgG production and a significant improvement in survival rates (Fig. 9E and F) [123].

In recent years, substantial progress has been made in vaccine development, particularly in the refinement of traditional platforms, including whole-cell, subunit, and conjugate vaccines. Concurrently, breakthroughs in nanoparticle delivery systems and novel adjuvant technologies have positioned nanovaccines at the forefront of immunological research. Among these, mucosal vaccines—especially those administered via the respiratory tract—have attracted significant interest due to their distinct immunological mechanisms and practical advantages.

A key strength of respiratory mucosal vaccines lies in their capacity to precisely elicit local immune responses at the initial sites of pathogen entry, such as the respiratory mucosa. By activating the mucosal immune system, these vaccines stimulate the production of SIgA within the lamina propria and promote the differentiation and persistence of CD4^+^ and CD8^+^ TRM cells. This multi-layered immune defense establishes a robust barrier at the portal of infection, offering the potential to achieve sterilizing immunity. Recent advances demonstrate that the integration of nanocarrier technology with innovative mucosal adjuvants—by enhancing antigen delivery efficiency—has led to notable improvements in the stability and immunogenicity of next-generation respiratory mucosal vaccine formulations.

Beyond their immunological advantages, mucosal vaccines also present practical benefits in production and regulatory compliance. Non-invasive delivery methods, such as nasal sprays, eliminate the risk of injection-related adverse events and potential transmission of bloodborne pathogens. This approach not only improves vaccine accessibility but also enhances public compliance, which is critical for mass vaccination campaigns. This relevance is amplified for pathogens like *KP* that invade via mucosal surfaces. The development of tailored mucosal vaccines promises comprehensive protection for both the upper and lower respiratory tract. These technological and immunological advances underscore the transformative potential of mucosal vaccines in future epidemic control and highlight their promising pathway toward clinical implementation.

## 5 Clinical translation and perspectives

Currently, no preventive or therapeutic vaccine against *KP* has been approved for global use. Although innovative approaches—such as reverse vaccinology and synthetic biology—have accelerated the development of vaccine candidates in recent years, clinical translation remains hindered by significant challenges. As a result, most candidate vaccines remain in the preclinical stage, with only a limited number progressing to clinical trials. This section reviews the current clinical progress and persistent obstacles in *KP* vaccine development.

### 5.1 Whole-cell vaccines

Current clinical research on *KP* whole-cell vaccines focuses predominantly on multivalent formulations. Among these, multivalent bacterial vaccines (MBVs) have emerged as a promising strategy to overcome the limitations of serotype-specific protection by integrating *KP* with other bacterial species such as *E. coli*. Clinical evidence from a pediatric infectious asthma trial demonstrated that an MBV containing heat-killed *Haemophilus influenzae*, *KP*, *Streptococcus pyogenes* (*S. pyogenes*), *S. aureus*, and *Streptococcus pneumoniae* (with additional *Neisseria catarrhalis*) induced hyposensitization. The low bacterial dosage was well-tolerated and significantly reduced the frequency and severity of infections. Critically, these effects were sustained for several years [76].

Similarly, Dentavax, composed of inactivated *KP*, *S. pyogenes*, *S. aureus*, *Candida albicans*, and *Lactobacillus acidophilus*, enhances systemic and mucosal immunity. Clinical data from volunteers indicated that it stimulated elevated serum IgG, salivary SIgA, and TNF-α production by peripheral blood lymphocytes [165]. Another MBV, Respivax, which includes inactivated *Streptococcus pneumoniae*, *Neisseria catarrhalis*, *S. pyogenes*, *Haemophilus influenzae* type B, *S. aureus*, and *KP*, was evaluated for respiratory infection prevention. Clinical trials showed that subjects with recurrent infections had reduced immune receptor expression at baseline. Following Respivax administration, significant upregulation of toll-like receptor 2 (TLR2), CD14, CD86, and human leukocyte antigen-DR isotype (HLA-DR) was observed in monocytes and polymorphonuclear leukocytes, along with restored IFN-γ and TNF-α levels comparable to healthy controls. No major adverse events were reported during the six-month follow-up [76,166]. Although these preparations, consisting of inactivated whole bacterial cells and lysates, represent a novel class of immunomodulatory agents, their precise mechanism of action and the relative contribution of each component remain to be fully elucidated. A critical next step will be to define these correlates, which is fundamental to guiding the rational development of next-generation polyvalent vaccines.

### 5.2 Subunit and recombinant vaccines

Recent advances in subunit and recombinant vaccine technologies have heightened interest in bacterial surface polysaccharides as promising antigenic targets. Among these, *KP'*s CPS is a particularly attractive target due to its high immunogenicity and surface accessibility, making it ideal for immune recognition.

Early foundational studies in murine and human models established that immunization with CPS induces robust, serotype-specific antibody responses [7]. This immunogenicity was confirmed in human trials during the 1980s. In these studies, vaccination with *KP*'s K1 CPS elicited significant IgG titers in healthy volunteers [7]. Building on this evidence, subsequent trials using K1 CPS alone or conjugated with *PA* components elicited high and durable IgM and IgG titers in healthy volunteers aged 16–59, with passive immunization from volunteer serum delaying mortality in burn-infected mice [76].

To broaden serotype coverage, vaccine development has increasingly focused on multivalent conjugate formulations. As a key example of this approach, a 24-valent *KP* CPS vaccine combined with an eight-valent *PA* OPS-toxin A conjugate vaccine elicited high antibody titers against 33 antigens (24 from *KP*, 9 from *PA*) for up to 18 months [167]. Moreover, in a study involving acute trauma patients (n = 10), the vaccine combination was well-tolerated, with only minor local tenderness reported in one individual and no systemic adverse events or hepatic abnormalities [168].

Clinical investigations of multivalent conjugate vaccines have paved the way for their advanced clinical development. This progress is particularly evident in *KP* vaccine research, where recent pivotal trials featuring enhanced target specificity and larger cohorts have demonstrated promising efficacy. Recent clinical advances highlight the tangible progress in *KP* vaccinology. The tetravalent glycoconjugate vaccine Kleb4V, targeting four critical *KP* O-serotypes (O1v1, O2a, O2afg, O3b), has successfully concluded a Phase I trial (NCT04959344). In a study of 166 healthy adults (including a safety cohort aged 18-40 years, n = 16, and a target cohort aged 55-70 years, n = 150), the vaccine demonstrated a favorable safety and immunogenicity profile. Critically, the vaccine elicited robust serotype-specific immune responses, which were significantly enhanced for three serotypes (O2a, O2afg, O3b) by the AS03 adjuvant [169]. In parallel, the field is exploring capsular targets. The bivalent K1/K2 glycoconjugate candidate CHO-V08 has entered Phase I trials (NCT07016152). Preliminary data from 20 healthy adults confirm its immunogenicity and favorable safety, showing dose-dependent increases in serotype-specific IgG titers without serious adverse events [170]. These advances demonstrate the efficacy of polysaccharide conjugate strategies in targeting distinct *KP* serotypes (O- and K-antigens), effectively addressing a critical gap in the vaccine landscape.

### 5.3 Persistent challenges and future directions

Despite encouraging progress, no *KP* vaccine has been approved, and significant challenges remain:

Antigenic Diversity: *KP* exhibits extensive serotype variation, with over 82 K-antigens and multiple O-antigens. Hypervirulent strains frequently express K1 or K2 capsules, while the O1, O2, O3, and O5 serotypes are clinically dominant. This diversity necessitates multivalent or broadly protective antigens, complicating vaccine design. Additionally, genetic recombination and point mutations can compromise vaccine-induced antibody efficacy. Therefore, achieving durable protection will require a dual approach: integrating continuous, genomic-driven surveillance with innovative antigen selection platforms to preemptively counter strain variation.

Immunogenicity Limitations: Polysaccharide conjugate vaccines function by converting TI antigens into TD antigens, thereby enhancing immunogenicity through the engagement of T-cell help. However, variations in conjugation efficiency can lead to inconsistent immune responses. Pre-existing immunity to carrier proteins (e.g., CRM197, diphtheria toxoid) may also interfere, especially in multivalent formulations. Developing novel carriers and improved conjugation techniques is essential for effective broad-spectrum vaccines.

Safety Concerns: Although current vaccines are generally safe, common adverse reactions include local redness, pain, and fever; severe allergic reactions are rare but possible. Given that the risk-benefit profile is not uniform across all individuals, safety considerations must be stratified by population. Individuals at the extremes of age, those who are immunocompromised, or patients with underlying autoimmune disorders may be at elevated risk, underscoring the necessity for population-specific safety assessments in future clinical development.

Lack of Mucosal Immunity: Injectable vaccines predominantly induce systemic IgG responses but typically induce weak mucosal SIgA responses, which limits their protection against mucosal colonization. To overcome this limitation, next-generation strategies are converging on the rational design of mucosal immunization. This entails the deployment of potent mucosal adjuvants, delivery systems (e.g., nanoparticles, sprays), and combined parenteral-mucosal immunization. The overarching goal of these integrated approaches is to orchestrate a synergistic interplay between systemic and local immunity, thereby achieving comprehensive protection.

## 6 Conclusions

This review highlights the evolving paradigm in *KP* vaccine development, shifting from serotype-specific strategies toward precision interventions that enhance mucosal immunity. The continued co-evolution of MDR and hypervirulent strains of *KP* underscores this pathogen's position on the WHO priority pathogen list. While antibiotics and passive antibody therapies offer transient relief, they underscore the critical need for robust active immunization strategies. Conventional injectable vaccines are constrained by antigenic diversity, capsule-mediated immune evasion, and LPS variability, redirecting focus toward mucosal vaccination-particularly via the respiratory tract, the primary site of bacterial colonization.

Targeting the respiratory mucosa enables activation of MALT, driving the production of SIgA to inhibit bacterial adhesion and iron acquisition, thereby establishing a critical first line of defense. Concurrently, this approach fosters the development of lung TRM cells, supporting durable immune surveillance and rapid recall responses.

Recent innovations include nanoscale biomimetic vesicles that enhance DCs targeting through surface functionalization while preserving antigen integrity and adjuvant activity. Structurally stabilized bacterial OMVs (BN-OMVs), encapsulated within nanogels, demonstrate improved *in vivo* stability and elicit high-titer, cross-protective antibodies following a single subcutaneous administration. At the molecular level, SpyTag/SpyCatcher-mediated covalent conjugation of conserved antigens (e.g., OmpK36, MrkA) to VLPs enables high-density epitope display, refining immunodominance and antibody specificity. Additionally, intranasal delivery of inactivated multivalent formulations is under investigation for its favorable safety profile and enhanced patient compliance, offering a standardized and scalable platform for next-generation mucosal *KP* vaccines.

Although multiple candidates, including multivalent polysaccharide conjugates, have advanced to clinical trials, challenges remain in achieving broad-spectrum efficacy, sustaining mucosal immunity, and scaling manufacturing processes. Future research should leverage synthetic biology tools—such as CRISPR-attenuated strains and AI-assisted antigen design—to refine inhalable vaccine platforms. Furthermore, elucidating the role of host-microbiota interactions in shaping immune memory will be crucial. With the growing integration of materials science and immunoengineering, *KP* vaccines are poised to transition from benchtop to bedside, providing a transformative strategy to limit the spread of drug-resistant infections.

## CRediT authorship contribution statement

**Jinping Hu:** Writing – original draft. **Yiyi Xie:** Writing – original draft. **Weiqi Guan:** Writing – original draft. **Linlin Huang:** Resources, Writing – original draft, Writing – review & editing. **Xin Li:** Resources, Writing – review & editing.

## Declaration of competing interest

The authors declare that they have no known competing financial interests or personal relationships that could have appeared to influence the work reported in this paper.

## Data Availability

No data was used for the research described in the article.
